# Normalizing sleep quality disturbed by psychiatric polypharmacy: a single patient open trial (SPOT)

**DOI:** 10.12688/f1000research.7694.2

**Published:** 2016-09-05

**Authors:** Victoria Magnuson, Yanpin Wang, Nicholas Schork

**Affiliations:** 1 Department of Human Biology, J Craig Venter Institute, La Jolla, CA, USA; 2 Decision Sciences,, First National Bank, Omaha, NE, USA; 3 Departments of Psychiatry, Family Medicine and Public Health, University of California, San Diego, CA, USA; 4 The Translational Genomics Research Institute, Phoenix, AZ, USA

**Keywords:** sleep, depression, polypharmacy, wireless monitoring, sleep apnea, personalized medicine, N-of-1 trials

## Abstract

There is a growing interest in personalized and preventive medicine initiatives that leverage serious patient engagement, such as those initiated and pursued among participants in the quantified-self movement. However, many of the self-assessments that result are not rooted in good scientific practices, such as exploiting controls, dose escalation strategies, multiple endpoint monitoring, etc. Areas where individual monitoring and health assessments have great potential involve sleep and behavior, as there are a number of very problematic sleep and behavior-related conditions that are hard to treat without personalization. For example, winter depression or seasonal affective disorder (SAD) is a serious, recurrent, atypical depressive disorder impacting millions each year. In order to prevent yearly recurrence antidepressant drugs are used to prophylactically treat SAD. In turn, these antidepressant drugs can affect sleep patterns, further exacerbating the condition. Because of this, possibly unique combinatorial or ‘polypharmaceutical’ interventions involving sleep aids may be prescribed. However, little research into the effects of such polypharmacy on the long-term sleep quality of treated individuals has been pursued. Employing wireless monitoring in a patient-centered study we sought to gain insight into the influence of polypharmacy on sleep patterns and the optimal course of therapy for an individual being treated for SAD with duloxetine (Cymbalta) and temazepam. We analyzed continuous-time sleep data while dosages and combinations of these agents were varied. We found that the administration of Cymbalta led to an exacerbation of the subject’s symptoms in a statistically significant way. We argue that such analyses may be necessary to effectively treat individuals with similar overall clinical manifestations and diagnosis, despite their having a unique set of symptoms, genetic profiles and exposure histories. We also consider the limitations of our study and areas for further research.

## Introduction

Winter depression or seasonal affective disorder (SAD) is an atypical depressive disorder that in most cases has onset in fall or winter with remission in spring or summer. It is estimated that approximately 5–10 percent of people in the U.S. (i.e., 10–20 million people) experience varying degrees of SAD in a given year ^1^. While full syndromal SAD (frequently dependent on additional external negative stressors) is not reached every year, subsyndromal symptoms can be seen ^2^. These symptoms are multiple, and include varying degrees of hypersomnia, carbohydrate-craving and jet-lagged physical and mental states (what is known as “brain fog”) resulting in fatigue and irritability. The annual shortening of the photoperiod is believed to be the main factor in SAD onset; however, responses to cold temperatures and epigenetic changes have been documented in seasonal mammals and exhibit evolutionary conservation down to lower forms of life ^3– 6^, suggesting that many very basic physiologic mechanisms could contribute to SAD. Ultimately, SAD is a complex disease with both chronobiological and neurobiological underpinnings ^7– 11^, which may include an etiology that for some could even begin in utero ^12– 16^.

Treating SAD is far from trivial and will require tailoring the treatment to an individual and his or her circumstances, for a whole host of reasons, not the least of which concern both individual and societal expectations regarding work habits, lifestyle, communal conventions surrounding day vs. nighttime activities, and the use of pharmacotherapies to treat conditions affecting behavior. In addition, SAD, and depressive syndromes in general, are known to be accompanied by many co-morbidities and sequelae, including anxiety, detrimental body habitus, anhedonia, and, more importantly, sleep disturbances which may exacerbate any underlying depression as well as the additional associated conditions ^2^. Tailored treatments for each and every condition possessed by an individual patient who also has SAD could adversely affect that patient’s sleep, thereby creating negative feedback for the SAD-related and other symptoms. Treatment of SAD includes a general recommendation for morning bright light therapy and/or antidepressant treatment which can be somewhat effective in managing symptoms, while melatonin, exercise and negative ion therapy are also suggested. However, a recent critical review of light therapy literature showed that most bright light therapy studies have methodological issues and evidence is not unequivocal ^17^. Further, cognitive response to bright light therapy can vary based on genetics ^18^. A proper prescription for light therapy requires knowing the dim light melatonin onset (DLMO) of SAD individuals (2/3 are phase-delayed) to determine circadian phase ^19^. The same is true for using supplemental melatonin to advance sleep phase, as improper timing and dosing can exacerbate symptoms ^19^. Because of the seasonal “on-off” nature of the disorder and difficulty in long-term compliance with bright light therapy (due to eyestrain and lack of individualized prescription), year-round prophylactic treatment with antidepressants may be prescribed.

Treatment for SAD and its sequelae are also compounded for peri- and post-menopausal females – a fact which may be under-appreciated in the primary care setting. The progression to menopause in normal women can result in circadian rhythm, vasomotor, and sleep disturbances and an increased risk for depression, possibly further exacerbating symptoms ^20– 22^. Therefore, a clinician’s choice to potentially increase the dosage of, e.g., a previously effective SSRI antidepressant can in turn exacerbate side effects, such as sleep disturbances. Importantly, sleep apnea is one of the most under-diagnosed conditions in post-menopausal women and is a leading cause of cardiovascular morbidity and mortality ^23– 29^. Prescribing sleep medications to aid in depression-related symptoms in peri- or post-menopausal women that may be susceptible, or have, sleep apnea is therefore highly problematic.

The fact that depression and sleep disturbances go hand in hand thus creates even more difficult treatment challenges. For example, ironically, it is known that many first-generation antidepressants exert their effects by, among other things, restoring sleep. Unfortunately, many second-generation antidepressants disrupt sleep. It is now accepted that SSRIs and SNRIs typically used to treat SAD can cause sleep disturbances, both in sleep quality (sleep initiation and maintenance) and sleep architecture (rapid eye movement (REM) and non-REM (NREM) sleep) ^30– 35^. Further, these agents can induce or escalate parasomnias such as periodic leg movements (PLMs) and restless legs syndrome (RLS) ^36, 37^. These effects on sleep could further lead clinicians to routinely prescribe sleep medications to counter the stimulating effects of antidepressants, as was recommended for insomnia in patients taking fluoxetine ^38– 40^. However, sleep medications can have their own negative impacts on sleep quality and architecture, and are not recommended for maintenance use. Thus, the resulting polypharmacy used to treat SAD is usually pursued without regard to the timing or dosage of the drugs or concern for drug-drug interactions. This fact, combined with unique patient characteristics such as age, gender, genetic and exposure profile, and co-morbid conditions, can further impact response to any prescribed drug or drug combination and may change over time.

In order to combat these issues, the management of SAD and related psychiatric disorders should, as noted, be pursued in a more patient-specific or ‘personalized’ manner – something that might not be accomplished at the level of a primary care provider. How such personalization can be achieved generally is an open question given the costs associated with the extra time a clinician might have to spend with a patient to determine an optimal course of therapy, but does suggest a greater number of empirical studies investigating the effects of polypharmacy and the utility of different treatment strategies are needed. In addition, patient-acceptance of the challenges surrounding treatment may motivate self-assessments of the type being pursued by members of the quantified self movement but perhaps in more objective ‘N-of-1’ clinical trial like settings ^41, 42^. We describe a study investigating the influence of polypharmacy involving a 58-year-old post-menopausal female who was diagnosed with SAD in 2001. The N-of-1 trial design utilized is known as a “single patient open trial” or SPOT ^41^. The SPOT offers an alternative to the typical N-of-1 trial components. A SPOT requires no randomization, no placebo and no blinding and allows limited cross-overs of one or more. The ultimate goals of the study were two-fold: to determine if objective claims about the influence of her treatments on her psychological well-being could be made in a self-assessment-oriented but designed outcome measures study, and whether her medication use correlated with exacerbation of her various symptoms and conditions.

Ultimately, the study leveraged wireless monitoring devices and regression modeling to assess patient sleep quality (e.g., the Zeo Sleep Monitor ^43, 44^), and designed a drug removal and dose escalation study to determine drug effects. In the course of the study, a number of important insights were obtained. The study identified a number of statistically significant correlations between medication use and symptomology that led to a number of potential recommendations for future treatments. Although it is important to acknowledge the shortcomings of the study, we feel that such patient-engaged and initiated yet protocol-oriented and designed N-of-1 studies may be the best way to individualize treatments for individuals with multiple mood and sleep-related conditions for which polypharmaceutical interventions are common.

## Methods

### Participant

We studied a post-menopausal 58-year-old female (the ‘subject’, author VLM) treated for SAD since 2001. The subject was interested in self-monitoring and an N-of-1 study for her sleep disturbances given her lengthy dissatisfaction with available treatment options, lack of insights into her multiple conditions, and a very elaborate and complex treatment history. The subject had a long history of usage of benzodiazepine as a sleep medication while taking antidepressants. The subject loosely qualifies as evening prone or delayed sleep phase disorder according to Basic Language Morningness Scale (BALM) questionnaire, which uses a 6-item scale ^45^. In summer 2012, she reported that under prolonged indoor low-light conditions she was susceptible to feeling fatigued, exhibiting seasonal symptomology even in summer months in San Diego. In fall 2012, the subject was taking 60 mg Cymbalta, 30 mg temazepam for sleep, and 100 mg sumatriptan as needed for morning headaches. An N-of-1 (SPOT design) study was pursued to explore how her medications affected her sleep in the context of her diagnosed winter depression (SAD), evening chronotype, delayed sleep phase, restless legs/PLMs and morning headaches.

### Ethics

The present study was self-administered by one of the authors (VLM). Therefore, ethical approval from an Institutional Review Board was not sought because the Helsinki Declaration does not apply in this case.

### Measures and wireless devices

***Sleep and activity monitoring.*** To assess sleep patterns a Zeo Sleep Monitor ( http://www.myzeo.com, model number ZEO 301) was used, which was worn nightly after entering bed per manufacturer instructions. The Zeo wirelessly tracks sleep stages at 5-minute intervals and has been validated against laboratory polysomnography ^43^. The number of awakenings (after sleep onset), percent time in light, deep, REM and wake were recorded and assessed with an accompanying iPAD application (Zeo Sleep Manager v1.9.0). Until the manufacturer’s bankruptcy, the Zeo online application provided nightly tracking of sleep stages and tools for evaluating trends. In addition, educational materials reminding the user of good sleep hygiene practices and journaling and counseling options were also offered. The data obtained with the Zeo monitor was captured on an iPad and Zeo graphic image data obtained with the device is available from the authors. In addition to the Zeo monitor, an Actiwatch Spectrum (manufactured by Philips Respironics) was used to collect data at 15-second intervals and worn daily to track sleep and light exposure. It was synchronized to the Zeo monitor on the nights it was worn. Because Actiwatch relies on movement to score wake versus sleep, the Actiwatch tends to overestimate time in sleep and underestimate time resting in a quiet awake state (Actiware software version 04.00). Periodic leg movements were measured using the PAM-RL (also manufactured by Philips Respironics) right and left ankle sensors and scored using default settings in software (PAM-RL version 7.6.2). Finally, the Fitbit Ultra actigraphic monitor ( http://www.fitbit.com) was worn daily to track walking or “step” activity. The subject wore the Fitbit on her waist from the start of her day through the evening. The Fitbit can be used to monitor sleep activity, but may overestimate sleep time since it keys off of movement (Fitbit app v1.8.2).

### Procedures

***Pharmacotherapy manipulation: effect on sleep.*** A schedule was developed for evaluating the effects of Cymbalta, temazepam and melatonin on the subject. Fourteen trials were conducted from 12-30-2012 through 07-05-2013. Description of the 14 trials and the number of nights with complete data are presented in the Results. Essentially, Cymbalta and temazepam were provided to the subject in pre-specified time periods with pre-specified doses initiated on weekends. Melatonin (Nature Made, 3 mg chocolate melts) was used to attempt to phase-shift the subject as needed to keep a work schedule, but several periods involving different combinations were pursued to explore the influence of melatonin on phase. Consistent with a SPOT design by definition and rationale, the study was pursued without randomization in a real-time, real-life setting, similar to a clinical practice drug de-escalation/withdrawal, and no medication blinding was utilized. In addition, because of the strong effects of the medications on our subject any placebo would have been detected. Similarly, a “no treatment washout period” between treatments was not employed or even feasible. There are several reasons for this, first, not wanting to destroy the continuity of the biological effects; but second, and more importantly, complete Cymbalta withdrawal causes undesirable side-effect symptoms such as “brain-zaps” for several months, the duration of which cannot be predicted. Hence in this case, washouts designed into this type of study would extend the timetable while causing further harms. We accept that this would add carryover and rebound effects at treatment boundaries. As an underlying goal of the study was to eliminate the benzodiazepine temazepam and to determine if any combination of Cymbalta and/or melatonin could normalize our subject’s sleep, we took an adaptive approach for which treatment cross-overs were only included in the latter portion of the study. It should also be noted that in designing a study like the one described there are a number of potential confounding variables that inevitably arise especially in any naturalistic, free-living setting assessing sleep quality: a) sleep consolidation could occur as sleep deprivation leads to sleep pressure as week progresses; b) sleeping in and changing sleep patterns on weekends could affect weekday trends; and c) percent time in wake after sleep onset can be increased by PLMs, sleep apnea or other sleep maintenance problems, which could be compounded by medication use.

***Sleep analysis.*** Each night and morning, the subject manually entered start and stop times into the Zeo sleep monitor iPAD app. The time to REM sleep was manually calculated based on Zeo graphic histogram output showing first REM sleep bar. Percent wake, light, deep and REM sleep and number of awakenings were supplied by the Zeo device. We did not use the Zeo sleep latency parameter “Time to Z” due to the confounding presence of PLMs, which our subject has shown to exhibit upon sleep initiation (clinically validated via videotape). The subject also wore the Actiwatch Spectrum around the clock from April 2013 until August 2013 as well as the PAM-RL ankle sensors nightly from April 2013 to July 2013. Some missing sleep quality data occurred due to days for which the subject was traveling.

### General statistical analysis

All analyses were performed using R version 3.1.3 ( http://www.R-project.org). For the sleep analysis, the data used contained information for 188 consecutive nights from December 30, 2012 to July 5, 2013 with 21 nights having missing data attributable to lost records and was therefore treated as missing at random (MAR). The response variables focusing on sleep quality included the number of wakes, time to first REM sleep, percent time in REM sleep, percent time in deep sleep, percent time in light sleep, and percent time in wake. To accommodate the presence of serial correlation in the nightly data, linear models considering an autoregressive moving average (ARMA) serial correlation structure among the data were fit. Different assumptions about the degree of serial correlation were made and tested. Interestingly, little evidence for a strong serial correlation was found, and therefore simple univariate linear regressions were used for all response variables via the lm function in R, retaining predictor variables significant at p < 0.05. Analyses involving model residuals were pursued to assess goodness-of-fit and satisfaction of linear model criteria. These included a Durbin-Watson test (to detect serial correlation between residual values), Shapiro-Wilk normality check, Portmanteau test and ARCH test. In cases where residuals in final models did not satisfy normality, a Box-Cox procedure was performed on the model. The resulting optimal exponential transformation was applied to the response variable and the model refit. To determine best fit among similar models, linear regression model fit measures (Akaike information criteria (AIC), Bayesian information criteria (BIC) and log likelihood) were evaluated. Only the best final models meeting all linear model criteria including no serial correlation or autocorrelation are presented in the results. The univariate regression models for each dependent variable were pursued in very similar ways, as outlined in the following example. Let perstage _*t*_denote series analysis response variables, where non-transformed variables are percent wake (perwake), percent light (perlight), percent deep (perdeep) and percent REM (perrem).

### Mathematics

To be more specific, an example model for perstage _*t*_ was created to follow the simple scheme below, with other variables leveraging similar models:

perstage _*t*_ = *μ*_0_ + *β*_*cym*30_ ∗ *cym*30 + *β*_*cym*60_ ∗ *cym*60 + *β*_*mel*3_ ∗ *mel*3 + *β*_*cym*30 *mel*3_ ∗ *cym*30 *mel*3 + *β*_*cym*30 *mel*6_ ∗ *cym*30 *mel*6 + *β*_*cym*60 *mel*3_ ∗ *cym*60 *mel*3 + *β*_*cym*60 *mel*6_ ∗ *cym*60 *mel*6 + *β*_*cym*60 *tem*15_ ∗ *cym*60 *tem*15 + *β*_*cym*60 *tem*30_ ∗ *cym*60 *tem*30 + *∈*_*t*_

where *μ*_0_ is a y-intercept term, the *β* terms are regression coefficients, *∈ _t_* is an error term with 0 mean and variance *σ* ^2^. The other terms in the model correspond to the drugs being evaluated and are denoted as follows: Cymbalta 30 mg (cym30); Cymbalta 60 mg (cym60); Melatonin 3 mg (mel3); Cymbalta 30 mg and Melatonin 3 mg (cym30mel3); Cymbalta 30 mg and Melatonin 6 mg (cym30mel6); Cymbalta 60 mg and Melatonin 3 mg (cym60mel3); Cymbalta 60 mg and Melatonin 6 mg (cym60mel6); Cymbalta 60 mg and Temazepam 15 mg (cym60tem15); Cymbalta 60 mg and Temazepam 30 mg (cym60tem30). Significant terms (i.e., p < 0.05 based on t-test of the coefficient value and its standard error) in the model were evaluated in an overall model fit as well as in a step-wise manner. Models were also fit to assess the impact of study design (night in time course) and days of the week (using Sunday as comparator per convention) by including these factors as independent variables in the model. The same analyses were performed for time to REM sleep.

## Results

Drug dosage and sleep response data Group = Drug trials, TTOREM = Time to REM sleep in hours, PERWAKE = Percent time in Wake, PERREM = Percent time in REM sleep, PERLIGHT = Percent time in Light sleep, PERDEEP = Percent time in Deep sleep, NWAKES = Number of wakes per night, DAY = Day of the week, DAYCODE = numerical code for day of the week, DAYSUNDAY, DAYMONDAY, etc. = contrast data codes, CYMDOSE, MELDOSE, TEMDOSE = dosing codes for Cymbalta, Melatonin, Temazepam, respectively, Cut-group = Cymbalta dose groups for plotting, DATE = Date of medication doses and night of sleep data collection, CYM30 = Cymbalta 30 mg, CYM60 = Cymbalta 60 mg, MEL3 = Melatonin 3 mg, MEL6 = Melatonin 6 mg, TEM15 = Temazepam 15 mg, TEM30 = Temazepam 30 mg, CYM30MEL3 = Cymbalta 30 mg and Melatonin 3 mg, CYM30MEL6 = Cymbalta 30 mg and Melatonin 6 mg, CYM60MEL3 = Cymbalta 60 mg and Melatonin 3 mg, CYM60MEL6 = Cymbalta 60 mg and Melatonin 6 mg, CYM60TEM15 = Cymbalta 60 mg and Temazepam 15 mg, CYM60TEM30 = Cymbalta 60 mg and Temazepam 30 mg, NA = missing data. Click here for additional data file. Copyright: © 2016 Magnuson V et al. 2016 Data associated with the article are available under the terms of the Creative Commons Zero "No rights reserved" data waiver (CC0 1.0 Public domain dedication).

PAM-RL Periodic Leg Movement Rates Sleep Date = Night of PLM collection, PLMs per hour Right ankle = Periodic leg movement counts per hour scored by PAM-RL software from right ankle sensor, PLMs per hour Left ankle = Periodic leg movement counts per hour scored by PAM-RL software from left ankle sensor. Click here for additional data file. Copyright: © 2016 Magnuson V et al. 2016 Data associated with the article are available under the terms of the Creative Commons Zero "No rights reserved" data waiver (CC0 1.0 Public domain dedication).

### Sleep quality analyses

Sleep data was collected for 188 consecutive nights from December 30, 2012 to July 5, 2013, with 21 nights having missing data ( Dataset 1). A description of the 14 trials and the number of nights with complete data are listed in Table 1 (abbreviations: Cymbalta (CYM); temazepam (TEM); melatonin (MEL)). Table 2 gives a descriptive analysis of the sleep parameters used in the study. The mean and standard deviation (SD) for: the number of times per night the subject was awakened (wakes (N)); time to first REM sleep bout in hours (1 ^*st*^ REM (h)); and percentage of time in each sleep stage (wake (%), light (%), deep (%), REM (%)) at each drug dose is shown. The number of days per dose and percent of the total nights are also shown (N days (%)). The dataset was not balanced in the sense that we had different numbers of observations while the subject was on different dosages of a drug.

**Table 1.** Drug trials and number of nights with complete data.

Trial	Start date	Nights	CYM	TEM	MEL
A	12-30-12	11	60 mg	30 mg	0 mg
B	01-11-13	7	60 mg	15 mg	0 mg
C	01-19-13	25	60 mg	0 mg	0 mg
D	02-13-13	9	60 mg	0 mg	3–6 mg
E	02-28-13	25	30 mg	0 mg	3–6 mg
F	03-27-13	10	30 mg	0 mg	3 mg
G	04-06-13	17	30 mg	0 mg	0 mg
H	04-24-13	7	30 mg	0 mg	3 mg
I	05-02-13	11	30 mg	0 mg	0 mg
J	05-17-13	14	30 mg	0 mg	3 mg
K	06-01-13	3	0 mg	0 mg	3 mg
L	06-04-13	7	0 mg	0 mg	0 mg
M	06-12-13	4	0 mg	0 mg	3 mg
N	06-16-13	17	0 mg	0 mg	0 mg

**Table 2.** Descriptive analysis of drug response variables.

Drug dose	Response variable	Cymbalta mean (SD)	Melatonin mean (SD)	Temazepam mean (SD)
0	Wakes (N) 1 ^*st*^ REM (h) Wake (%) Light (%) Deep (%) REM (%)	7.03 (2.40) 1.26 (0.51) 9.00 (7.77) 33.68 (6.37) 21.48 (4.19) 35.81 (4.97)	12.67 (4.66) 3.20 (1.56) 16.08 (9.36) 42.66 (9.72) 15.20 (6.26) 25.98 (8.55)	13.11 (4.64) 3.23 (1.38) 18.68 (10.39) 38.48 (6.27) 16.11 (4.29) 26.68 (7.43)
	N days (%)	31 (18.56%)	95 (56.89%)	149 (89.22%)
1	Wakes (N) 1 ^*st*^ REM (h) Wake (%) Light (%) Deep (%) REM (%)	14.81 (3.80) 3.53 (0.93) 20.63 (9.74) 38.81 (5.46) 14.80 (3.15) 25.70 (5.49)	13.79 (4.20) 3.30 (1.15) 20.67 (10.08) 37.41 (6.20) 15.28 (3.44) 26.66 (5.89)	13.86 (1.86) 4.14 (0.46) 17.43 (4.83) 57.57 (3.05) 7.71 (1.60) 17.29 (4.23)
	N days (%)	84 (50.30%)	58 (34.73%)	7 (4.19%)
2	Wakes (N) 1 ^*st*^ REM (h) Wake (%) Light (%) Deep (%) REM (%)	14.13 (3.17) 4.15 (1.23) 20.50 (8.39) 47.65 (9.32) 11.42 (4.89) 20.44 (5.69)	13.79 (4.14) 3.97 (1.10) 25.00 (9.81) 39.93 (5.58) 12.36 (2.50) 22.71 (5.97)	13.27 (3.35) 4.14 (0.46) 15.55 (6.68) 58.73 (5.39) 4.45 (1.57) 21.36 (6.22)
	N days (%)	52 (31.14%)	14 (8.38%)	11 (6.59%)

**Figure 1.**
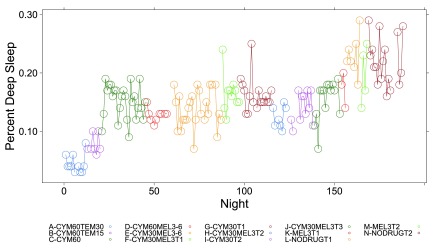
Percent deep sleep per night. The percent time subject was in deep sleep during 5-minute intervals detected by the Zeo Sleep Monitor throughout the entire study. Dosages of Cymbalta (CYM60 = 60 mg, CYM30 = 30 mg), temazepam (TEM30 = 30 mg, TEM15 = 15 mg) and melatonin (MEL3 = 3 mg, MEL6 = 6 mg) were varied according to combinations A–N (T1, T2, T3 are trial replicates), including no drug trials (L, N).

**Figure 2.**
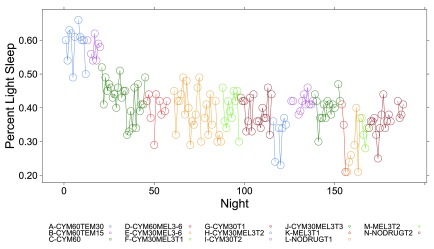
Percent light sleep per night. The percent time subject was in light sleep during 5-minute intervals detected by the Zeo Sleep Monitor throughout the entire study. Dosages of Cymbalta (CYM60 = 60 mg, CYM30 = 30 mg), temazepam (TEM30 = 30 mg, TEM15 = 15 mg) and melatonin (MEL3 = 3 mg, MEL6 = 6 mg) were varied according to combinations A-N (T1, T2, T3 are trial replicates), including no drug trials (L, N).

**Figure S1.**
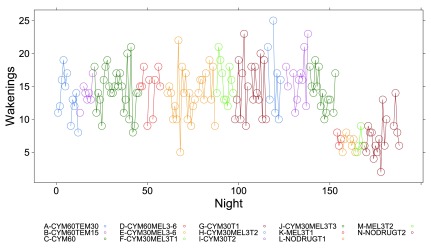
Wakenings per night. The number of times per night subject was awake during 5-minute intervals detected by the Zeo Sleep Monitor throughout the entire study. Dosages of Cymbalta (CYM60 = 60 mg, CYM30 = 30 mg), temazepam (TEM30 = 30 mg, TEM15 = 15 mg) and melatonin (MEL3 = 3 mg, MEL6 = 6 mg) were varied according to combinations A-N (T1, T2, T3 are trial replicates), including no drug trials (L, N).

**Figure S4.**
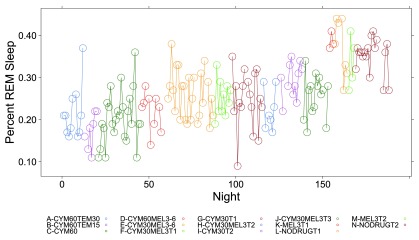
Percent REM sleep per night. The percent time subject was in REM sleep during 5-minute intervals detected by the Zeo Sleep Monitor throughout the entire study. Dosages of Cymbalta (CYM60 = 60 mg, CYM30 = 30 mg), temazepam (TEM30 = 30 mg, TEM15 = 15 mg) and melatonin (MEL3 = 3 mg, MEL6 = 6 mg) were varied according to combinations A-N (T1, T2, T3 are trial replicates), including no drug trials (L, N).

Figure 1, Figure 2 and Figure S1– Figure S4 (see Supplementary Material) graphically depict the impact of Cymbalta, melatonin and temazepam drug use on the subject’s sleep architecture. Figure 1 and Figure 2 show the percent of time per night that the subject was in deep sleep and light sleep, respectively, during 5-minute intervals detected by the Zeo Sleep Monitor throughout the entire study. Similar figures for the number of times the subject was awakened, time to REM sleep, percent time after sleep onset that the subject was awake and percent time in REM sleep during 5-minute intervals detected by the Zeo Sleep Monitor are presented in the Supplementary Material ( Figure S1– Figure S4, respectively).

**Figure 3.**
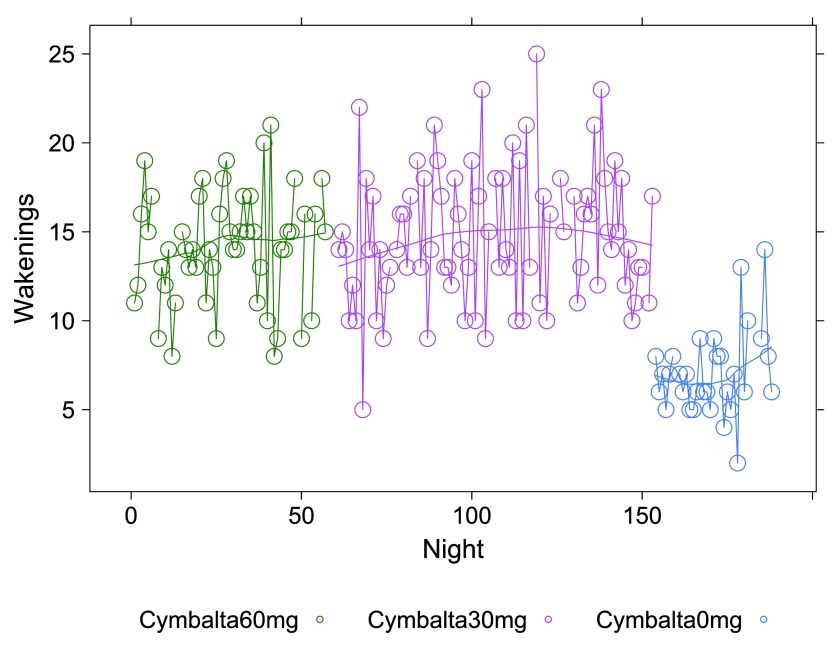
Wakenings per night by Cymbalta dose. The number of times per night subject was awake during 5-minute intervals detected by the Zeo Sleep Monitor. Doses of Cymbalta were decreased from 60 mg to 0 mg.

**Figure 4.**
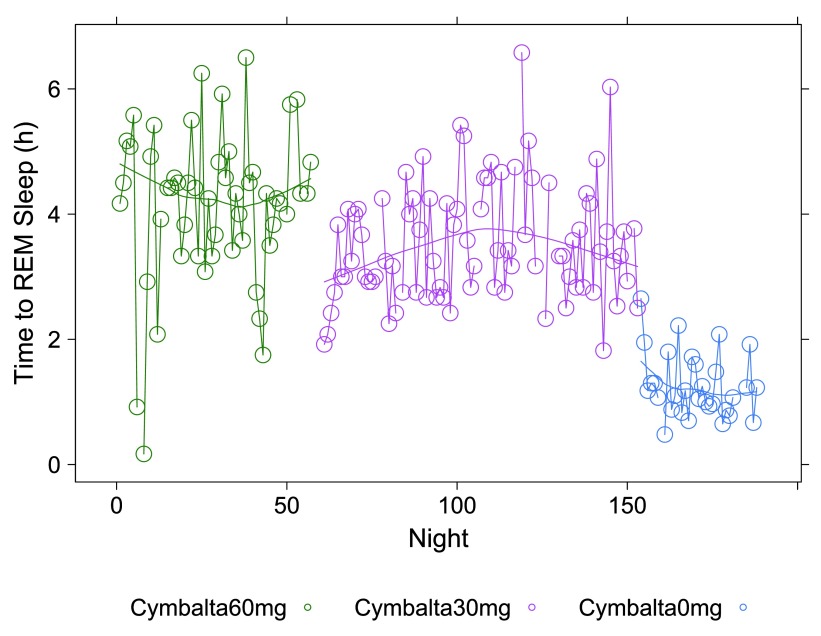
Time to REM sleep per night by Cymbalta dose. The number of hours (h) per night before subject achieved first REM sleep bout during 5-minute intervals detected by the Zeo Sleep Monitor. Doses of Cymbalta were decreased from 60 mg to 0 mg.

**Figure 5.**
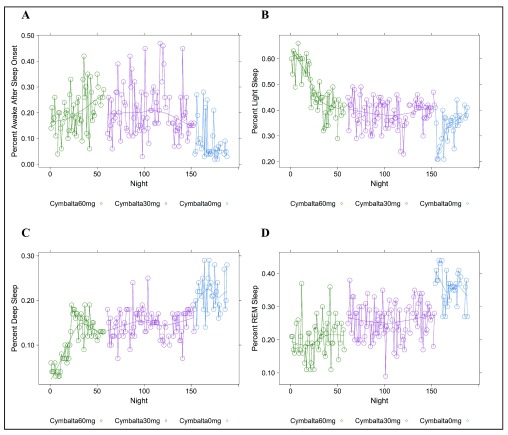
Percent time in wake, light, deep and REM sleep per night by Cymbalta dose. Percent time after sleep onset subject was awake ( **A**); subject was in light sleep ( **B**); subject was in deep sleep ( **C**); or subject was in REM sleep ( **D**) during 5-minute intervals detected by the Zeo Sleep Monitor. Doses of Cymbalta were decreased from 60 mg to 0 mg.

A clear relationship can be seen between temazepam intake and reduced deep sleep in favor of light sleep ( Figure 1 and Figure 2). However, Cymbalta had the strongest impact on the subject’s sleep architecture as shown in Figure 3, Figure 4, and Figure 5. Cymbalta intake increased the number of awakenings ( Figure 3), time to first REM sleep ( Figure 4), percent time after sleep onset that subject was awake (wake) ( Figure 5A) and in light sleep ( Figure 5B) at the expense of deep ( Figure 5C) and REM ( Figure 5D) sleep. Removal of Cymbalta decreased the number of awakenings, time to first REM sleep, percent time in wake and light sleep and increased percent time in deep and REM sleep ( Figure 3– Figure 5).

Because of the free-living nature of our study, the subject’s polypharmacy and struggle to counter sleep disturbances, a large variability in the data is seen. In addition, “normal” sleep staging typically follows a pattern wherein the first non-REM sleep (light plus deep sleep) and REM sleep cycle is completed in 70 to 100 minutes, followed by 90 to 120 minute cycles, with deep sleep bouts gradually disappearing and REM sleep bouts lengthening throughout the night ^46^. Near the end of the night, usually only light and REM sleep periods make up the sleep cycles. As a result, we chose to analyze the percentage of time the subject was in each sleep/wake state, rather than total time. For the purposes of comparing Zeo monitored stages to classically defined sleep stages, we assumed the following to represent approximately “normal” sleep stage percentages: wake 5 percent; light 45–55 percent; deep 20–25 percent; REM 25 percent ^46^.

Table 3 summarizes the results of our univariate analyses when the sleep stages, wake, light, deep and REM, were taken as dependent variables. The univariate linear regression models were performed as described (see Methods) and data is presented as mean percent for each sleep stage with treatment effects adjusted relative to the intercept. Analyses of percent wake and light sleep met Durbin-Watson test criteria once two outlier nights each were removed. Final model diagnosis showed that all linear regression assumption requirements were satisfied except for the normality condition for percent wake and percent light sleep. Therefore, the Box-Cox procedure and transformations were performed and the models refit. Final models satisfied all diagnostic tests and the transformed mean estimate values (denoted as ‘bc’) presented in Table 3 were adjusted and back-transformed to give mean percent wake and light sleep.

**Table 3.** Univariate regression analysis predicting percent wake, light, deep and REM sleep from drug and dose, as well as day of the week, information.

Variable	Mean (%)	Wake estimate ^*bc*^ (SE)	p-value	Mean (%)	Light estimate ^*bc*^ (SE)	p-value	Mean (%)	Deep estimate (SE)	p-value	Mean (%)	REM estimate (SE)	p-value
*μ*_0_	10.5	0.39 (0.02)	< 2e-16	35.4	0.16 (0.01)	< 2e-16	22.3	0.22 (0.01)	< 2e-16	34.2	0.34 (0.01)	< 2e-16
*β*_*cym*30_	20.8	0.13 (0.02)	3.2e-07	40.0	0.04 (0.01)	0.0015	15.4	-0.07 (0.01)	7.7e-14	24.8	-0.09 (0.01)	5.7e-11
*β*_*cym*60_	25.3	0.17 (0.02)	6.3e-11	43.1	0.07 (0.01)	1.8e-07	15.0	-0.07 (0.01)	3.3e-14	18.7	-0.15 (0.01)	< 2e-16
*β*_*mel*3_	—	—	—	—	—	—	18.7	-0.04 (0.01)	0.0068	—	—	—
*β*_*cym*30 *mel*3_	25.0	0.17 (0.02)	3.4e-13	38.4	0.02 (0.01)	0.0201	15.0	-0.07 (0.01)	< 2e-16	23.6	-0.11 (0.01)	1.1e-15
*β*_*cym*30 *mel*6_	25.8	0.18 (0.03)	4.0e-07	39.7	0.03 (0.02)	0.0358	12.2	-0.10 (0.01)	2.0e-15	23.8	-0.10 (0.02)	1.0e-07
*β*_*cym*60 *mel*3_	31.0	0.22 (0.04)	1.3e-06	—	—	—	12.8	-0.09 (0.01)	2.1e-09	22.2	-0.12 (0.02)	2.9e-06
*β*_*cym*60 *mel*6_	30.6	0.22 (0.05)	1.4e-05	41.5	0.05 (0.02)	0.0356	12.8	-0.10 (0.02)	3.3e-08	18.1	-0.16 (0.03)	1.9e-08
*β*_*cym*60 *tem*15_	20.9	0.13 (0.04)	0.0010	57.6	0.22 (0.02)	< 2e-16	7.7	-0.15 (0.01)	< 2e-16	15.8	-0.18 (0.02)	1.0e-14
*β*_*cym*60 *tem*30_	17.8	0.10 (0.03)	0.0034	58.9	0.23 (0.02)	< 2e-16	4.5	-0.18 (0.01)	< 2e-16	20.5	-0.14 (0.02)	2.5e-12

*β _Night_*	—	—	—	—	—	—	—	—	—	—	—	—
*β _Weekday_*				—	—	—	—	—	—			
*β _Monday_*	—	—	—			—	—	—
*β _Tuesday_*	7.1	-0.06 (0.02)	0.0123		—	—	—
*β _Wednesday_*	7.4	-0.05 (0.02)	0.0177		—	—	—
*β _Thursday_*	6.5	-0.07 (0.02)	0.0017							37.0	0.03 (0.01)	0.0154
*β _Friday_*	7.3	-0.05 (0.02)	0.0239							37.9	0.04 (0.01)	0.0029
*β _Saturday_*	4.8	-0.11 (0.02)	1.5e-05							39.6	0.05 (0.01)	3.1e-05

	bc:exponent = 0.42			bc:exponent = 1.79				
	Adjusted R2: 0.3900			Adjusted R2: 0.6604			Adjusted R2: 0.6629			Adjusted R2: 0.5460		
	p-value: 1.3e-13			p-value: < 2.2e-16			p-value: < 2.2e-16			p-value: < 2.2e-16		

Adjusted mean in percent, mean estimate or transformed mean estimate ^*bc*^, standard error (SE) and p-value (Pr > |t-value|). R2: R-squared; bc: Box-Cox transformed variable raised to exponent given in final model. Back-transformation to original units was performed (after adjustments relative to intercept) by taking the nth (exponent) root of estimate.

From Table 3 it is clear many of the drugs, doses and drug combinations have a highly significant and negative impact on deep and REM sleep (with the exception of melatonin at 3 mg). The estimate of the y-intercept ( *μ*_0_) for the model with deep sleep as the dependent variable suggests that approximately 22.3 percent of the time the subject was in deep sleep without any drug effects (p < 2×10 ^-16^). The estimated coefficients for the drug and drug dosage independent variables in the model provide the effect on deep sleep of the drugs. The mean percent deep sleep ranged from 4.5 percent (-0.18 (SE: 0.01), p < 2×10 ^-16^) while the subject was taking 60 mg Cymbalta and 30 mg temazepam to 18.7 percent (-0.04 (SE: 0.01), p = 0.0068) while the subject was taking 3 mg melatonin. Although temazepam dosing in combination with Cymbalta had the greatest negative impact on deep sleep in favor of light sleep, Cymbalta alone continued to interfere with deep sleep.

Similarly, the impact of an antidepressant such as Cymbalta is expected to show a decrease in REM sleep, mainly through the delay in REM sleep onset (see Table 4). The estimate of the y-intercept ( *μ*_0_) for the model with REM sleep as the dependent variable suggests that approximately 34.2 percent of the time the subject was in REM sleep without any drug effects (p < 2×10 ^-16^), which might be considered high compared to the usual 25 percent. The estimated coefficients for the drug and drug dosage independent variables in the model provide the effect on REM sleep of the drugs. The mean percent REM sleep ranged from 15.8 percent (-0.18 (SE: 0.02), p = 1×10 ^-14^) while the subject was taking 60 mg Cymbalta and 15 mg temazepam to 24.8 percent (-0.09 (SE: 0.01), p = 5.7×10 ^-11^) while the subject was taking 30 mg Cymbalta. Interestingly, there was an increase in REM sleep on Thursday, Friday and especially significant on Saturday (39.6 percent (0.05 (SE: 0.01), p = 3.1×10 ^-5^)).

**Table 4.** Univariate series analysis for predicting time to REM sleep.

Variable	Mean (h)	Time to REM sleep estimate ^*bc*^ (SE)	p-value
*μ*_0_	1.27	1.09 (0.03)	< 2e-16
*β*_*cym*30_	3.73	0.54 (0.04)	< 2e-16
*β*_*cym*60_	4.22	0.62 (0.04)	< 2e-16
*β*_*mel*3_	—	—	—
*β*_*cym*30 *mel*3_	3.55	0.51 (0.04)	< 2e-16
*β*_*cym*30 *mel*6_	3.43	0.49 (0.06)	8.1e-15
*β*_*cym*60 *mel*3_	4.33	0.64 (0.08)	1.8e-14
*β*_*cym*60 *mel*6_	5.16	0.76 (0.08)	4.9e-16
*β*_*cym*60 *tem*15_	4.27	0.63 (0.07)	< 2e-16
*β*_*cym*60 *tem*30_	4.43	0.64 (0.06)	< 2e-16

*β _Night_*	—	—	—
*β _Weekday_*				
*β _Friday_*	1.05	-0.08 (0.04)	0.0458
*β _Saturday_*	1.01	-0.09 (0.04)	0.0174

	bc: exponent = 0.375		
	Adjusted R2: 0.6823		
	p-value: < 2.2e-16		

Adjusted mean in hours (h), transformed mean estimate ^*bc*^, standard error (SE) and p-value (Pr > |t-value|). R2: R-squared; bc: Box-Cox transformed variable raised to exponent given in final model. Back-transformation to original units was performed (after adjustments relative to intercept) by taking the nth (exponent) root of estimate.

Most drug combinations, except melatonin, significantly increased time in wake and light sleep. Of note, the Zeo monitor can detect micro-arousals as well as conscious wakes. Thus, some scores of the wakes at night may actually be classified as light sleep. However, from Table 2 and Table 3 the drug combinations increase both of these at the expense of deep and REM. The effect of increasing light sleep at the expense of deep sleep is most notably seen with temazepam use. The estimate of the y-intercept ( *μ*_0_) for the model with light sleep as the dependent variable suggests that approximately 35.4 percent of the time the subject was in light sleep without any drug effects (p < 2×10 ^-16^). The estimated coefficients for the drug and drug dosage independent variables in the model provide the effect on light sleep of the drugs. The mean percent light sleep ranged from 38.4 percent (0.02 (SE: 0.01), p = 0.0201) while the subject was taking 30 mg Cymbalta and 3 mg melatonin to 58.9 percent (0.23 (SE: 0.02), p < 2×10 ^-16^) while the subject was taking 60 mg Cymbalta and 30 mg temazepam.

The major impact on wake after sleep onset occurred after the removal of temazepam and during Cymbalta use, indicating a possible sleep maintenance issue. The estimate of the y-intercept ( *μ*_0_) for the model with wake as the dependent variable suggests that approximately 10.5 percent of the time the subject was in wake without any drug effects (p < 2×10 ^-16^). The estimated coefficients for the drug and drug dosage independent variables in the model provide the effect on wake of the drugs. The mean percent wake ranged from 17.8 percent (0.10 (SE: 0.03), p = 0.0034) while the subject was taking 60 mg Cymbalta and 30 mg temazepam to 31.0 percent (0.22 (SE: 0.04), p = 1.3×10 ^-6^) while the subject was taking 60 mg Cymbalta and 3 mg melatonin. Interestingly, there is evidence for decreased time classified as wake as the week progresses that might be attributed to a number of things such as increasing sleep pressure during the week, relaxed frame of mind and sleeping in on the weekend. In fact, the decrease in wake to 4.8 percent on Saturday seems to approximately parallel the increase in REM sleep on Saturday (approximately 5 percent) with similar p-values. There was no impact of the night of the study on any of the models.

Table 4 shows the univariate analyses of time to REM sleep in hours as a dependent variable. The univariate linear regression model exhibited no serial correlation based on the Durbin-Watson test once two Zeo technical outlier nights were removed (known REML error ^43^). As above, models were also tested for the impact of study design (night in time course) and day of the week. An assessment of the normality and serial correlation among the residuals obtained from the model was performed by Portmanteau test, Durbin-Watson statistic, a standard normality check and ARCH test which showed that all linear regression assumption requirements were satisfied except normality. Therefore, the Box-Cox procedure and transformation was performed, and model refit as above. The mean estimates presented in Table 4 were adjusted and back-transformed to give the original unit of hours.

All drug combinations except for melatonin at the 3 mg dose caused large and highly significant increases in time to first REM sleep. Under normal circumstances the first REM bout is expected to occur before completing the first 70–100 minute full cycle of sleep (light + deep + REM), that is, in less than 2 hours. The estimate of the y-intercept ( *μ*_0_) for the model with time to first REM sleep as the dependent variable suggests that time to first REM sleep for the subject was 1.27 hours (76.2 minutes) without any drug effects (p < 2×10 ^-16^), which is in the correct range for the first full sleep cycle. The estimated coefficients for the drug and drug dosage independent variables in the model provide the effect on time to REM sleep of the drugs. The drug effects ranging from most to least deleterious impact on mean percent time to REM sleep are: Cymbalta 60 mg and melatonin 6 mg, 5.16h (0.76 (SE: 0.08), (p = 4.9×10 ^-16^); Cymbalta 60 mg and temazepam 30 mg, 4.43h (0.64 (SE: 0.06), (p < 2×10 ^-16^); Cymbalta 60 mg and melatonin 3 mg, 4.33h (0.64 (SE: 0.08), (p = 1.8×10 ^-14^); Cymbalta 60 mg and temazepam 15 mg, 4.27h (0.63 (SE: 0.07), (p < 2×10 ^-16^); Cymbalta 60 mg, 4.22h (0.62 (SE: 0.04), (p < 2×10 ^-16^); Cymbalta 30 mg, 3.73h (0.54 (SE: 0.04), (p < 2×10 ^-16^); Cymbalta 30 mg and melatonin 3 mg, 3.55h (0.51 (SE: 0.04), (p < 2×10 ^-16^) and Cymbalta 30 mg and melatonin 6 mg, 3.43h (0.49 (SE: 0.06), (p = 8.1×10 ^-15^). There was no impact of the night of the study on the model. Of note, is the decrease in time to REM sleep on weekend nights.

The data shows an unequivocal Cymbalta dose-response, decreasing the time to REM sleep with decreasing Cymbalta dose as expected. Even under the least damaging drug regimen, time to first REM sleep was still delayed over 1.75 hours compared to the maximum in normal sleep architecture (3.43 hours versus 1.67 hours or 100 minutes). This delay in first REM sleep could possibly push normal REM sleep cycling into later parts of the night and interfere with the ability to naturally wake the next morning. Further, truncating REM sleep while keeping a daily work-week schedule might be expected to have additional functional and metabolic consequences.

We used the data to attempt to predict a lower Cymbalta drug dose which might not be expected to interfere with our subject’s sleep or perhaps normalize all of the percent sleep stages toward “normal” ranges (i.e., wake 5 percent; light 45–55 percent; deep 20–25 percent; REM 25 percent ^46^) since our subject has increased REM (34 percent) and decreased light (35 percent) sleep if drug effects were accounted for. Table 5 provides the predicted values for 10 mg and 20 mg Cymbalta doses based on the fitted regression models.

**Table 5.** Predicted sleep architecture responses at intermediate Cymbalta doses.

Cymbalta dose	Time to REM hours	Percent wake	Percent light	Percent deep	Percent REM
0 mg	1.27	10.5	35.4	22.3	34.2
10 mg	2.09	13.9	36.9	20.0	31.1
20 mg	2.91	17.4	38.5	17.7	27.9
30 mg	3.73	20.8	40.0	15.4	24.8

We note that even considering the removal of Cymbalta altogether, the percentage of the sleep time our subject was estimated to be in a ‘wake’ period as detected by the Zeo monitor is high. PLMs that tracked with Cymbalta use did decrease to less than 15 per hour during the study (see Dataset 2) which is considered to be normal and therefore not likely to be a source of confusion for the Zeo monitor since episodes of PLMs may confound time in the wake period. However, micro-arousals and unconscious wakes due to the possible presence of mild sleep apnea in our subject remained a concern and could be reflected in the sleep values we observed. A follow-up study performed to monitor a clinical intervention to correct mild sleep apnea is presented in the Supplementary Material.

## Discussion

We have shown that monitoring an individual’s response to various drugs used to treat her severe sleep and sleep-related disturbances yielded important and actionable insights. For example, the subject’s sleep quality was highly compromised when taking Cymbalta at therapeutic (60 mg) and sub-therapeutic (30 mg) doses and was likely aggravated further by polypharmaceutical interventions she was prescribed. In addition, the subject’s other conditions, such as mild sleep apnea, may also have contributed to her sleep disturbances and general physical and psychological health. While sleep disruption is a common side effect of SSRIs and SNRIs, our finding that Cymbalta appears to have exacerbated the subject’s condition, is important for personalized care of patients with nuanced conditions. The problems associated with Cymbalta may have been due to the extended release formulation of the drug. It is known that Cymbalta is metabolized by CYP2D6, which has been recently shown to undergo a metabolizer phenotype conversion that cannot be assessed by genetic testing ^47^. Drug-induced and particularly co-medication-induced phenoconversion is an increasing problem for personalized medicine ^48^. Additionally, temazepam is not a short-acting benzodiazepine drug and can cause hangover effects in the course of a night that could contribute to the phase-delay our subject experienced. In fact, both temazepam and another highly used sleep aid, Ambien, were recently found to be associated with increased morbidity and mortality ^49^. Despite the fact our subject was co-morbid for a number of circadian disruptors, her sleep architecture normalized when all drugs were removed. In addition, drug removal unmasked mild sleep apnea, manifesting mainly during an NREM sleep component. The temazepam-Cymbalta combination appears to have induced a removal of deep sleep that actually mimics the shallow sleep architecture seen in depressed patients ^50^. Antidepressants are often touted as able to restore deep sleep and delay REM sleep in depression ^50^. However, for the subject of focus here (and we suspect many others), the major destruction of her deep sleep occurred when a sleep aid was added to counteract the over-stimulation of the antidepressant.

A number of studies have shown that antidepressants can exacerbate symptoms associated with depression ^30– 37^. Further, we found that our subject suffered from mild obstructive sleep apnea (OSA) and should probably never have been on sleep medication in the first place. Symptom clusters of poor sleep, migraines, and fatigue should motivate a physician to perform a sleep study. In fact, both in menopausal women and in psychiatric practice where mood and sleep disorders can show bi-directional causation, ordering sleep studies for patients has become the recommended course ^51, 52^.

### Limitations

The drug withdrawal protocol for the subject discussed here ran from December to July. The days were getting longer across the time period (after winter solstice to after summer solstice) so changes in the subject’s responses to light and increased/decreased internal secretion of melatonin/serotonin could have had a beneficial influence on the direction of the changes in sleep parameters in parallel with drug removal. Alternatively, the hypersomnia expected in a SAD-susceptible individual during December-May could result in a more sound sleep (except for sleep latency issues expected from her phenotype/chronotype). However, we showed that the final (no-drug) sleep architecture in July 2013 was equivalent to that observed at the beginning of our sleep apnea intervention in December 2013 (see Supplementary Material). In the end, the subject demonstrated what is typical for SAD, normal sleep architecture, but tendency toward a delayed chronotype.

Due to the free-living nature of our study, attempts to follow/collect standardized food, exercise and sleep/wake behavior were not maintained, although, attempts to phase-shift to earlier sleep/wake regimens were documented. Applying a SPOT design, there was no randomization, drug placebo, blinding or washouts between trials, but we were able to compare our subject’s status to her status at times when no drug was provided in a crossover setting. Abrupt changes in treatment may have contributed some expected and some unexpected noise to the data. For example, temazepam dose decreases would be expected to result in delayed sleep onset, however, changes from Cymbalta 60 mg to Cymbalta 30 mg caused hot-flashes also impacting sleep initiation. For the most part, we collected enough data under each treatment studied (relative to drug or device on/off) to measure effects, including the capture of rebound and recovery effects, and the duration of our individual trial conditions were comparable to what is often seen in sleep literature. As stated in the Methods, our decision was to use a real-time/real-life dose withdrawal and not to use washout periods (the appropriate duration of “washout” would be hard to determine for Cymbalta) to avoid harms. As it was, our Cymbalta dose de-escalation was slower than what is used in clinical practice (Cymbalta 60 mg for 52 days, Cymbalta 30 mg for 84 days, Cymbalta 0 mg for 31 days). We also limited the number of times any one drug combination was provided. Given the number of drugs and the number of doses studied, it would be virtually impossible to accommodate multiple intervals with the same drugs and dosages given. Again, given the strong impact of the drugs used in this case, as evidenced by the variability in the data, only the lower dose trials included cross-overs. This resulted in trials at the beginning of the study having only one measurement, albeit covering periods from 7–25 days each. A similar range in days is seen when the duration of the individual same drug/dose regimen trial replicates are combined.

## Conclusions

Many people suffering from circadian and sleep disturbances such as those found in SAD have very unique genetic determinants for their condition, different sets of sleep disturbance sequelae, secondary conditions, and nuanced lifestyles that make it hard to treat them exactly the same way. As a result, more focused attention on what intervention strategy makes the most sense to pursue is required. Such ‘personalized’ intervention strategies are not trivial to implement since they require an integrated, objective, and often-times completely empirical approach to identify and implement them. We describe our experience with, and the results of, a comprehensive investigation into the response of a single patient to designed manipulations of her sleep pharmacology. We find that the patient had underlying conditions (e.g., sleep apnea) that were confounded by the use of specific drugs to treat her SAD and that these drugs contributed to, or exacerbated, other issues in the subject’s life (e.g., alert time for work, attempts to make up for lack of quality sleep during the week on the weekends, etc.). Ultimately, our study and its results should set a precedent for patient-oriented, yet designed and objective, investigations into the impact of polypharmacy and general drug response in real-world settings.

## Data availability

The data referenced by this article are under copyright with the following copyright statement: Copyright: © 2016 Magnuson V et al.

Data associated with the article are available under the terms of the Creative Commons Zero "No rights reserved" data waiver (CC0 1.0 Public domain dedication). 

*F1000Research*: Dataset 1. Drug dosage and sleep response data, 10.5256/f1000research.7694.d112016 ^53^

*F1000Research*: Dataset 2. PAM-RL Periodic Leg Movement Rates, 10.5256/f1000research.7694.d130338 ^54^

## Consent

Written informed consent for publication of their clinical details and/or clinical images was obtained from the patient.

## Figures and Tables

**Figure S2.**
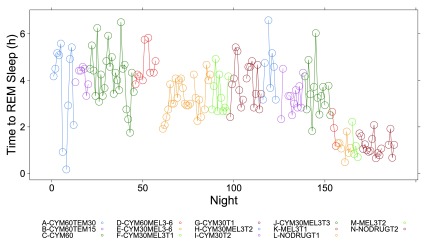
Time to REM sleep per night. The number of hours (h) per night before subject achieved first REM sleep bout during 5-minute intervals detected by the Zeo Sleep Monitor throughout the entire study. Dosages of Cymbalta (CYM60 = 60 mg, CYM30 = 30 mg), temazepam (TEM30 = 30 mg, TEM15 = 15 mg) and melatonin (MEL3 = 3 mg, MEL6 = 6 mg) were varied according to combinations A-N (T1, T2, T3 are trial replicates), including no drug trials (L, N).

**Figure S3.**
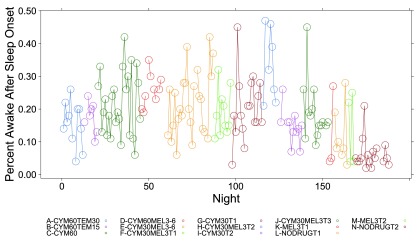
Percent time awake after sleep onset. The percent time subject was awake during 5-minute intervals detected by the Zeo Sleep Monitor throughout the entire study. Dosages of Cymbalta (CYM60 = 60 mg, CYM30 = 30 mg), temazepam (TEM30 = 30 mg, TEM15 = 15 mg) and melatonin (MEL3 = 3 mg, MEL6 = 6 mg) were varied according to combinations A-N (T1, T2, T3 are trial replicates), including no drug trials (L, N).

**Table S1.** Univariate series analysis for predicting HRV by mouth guard setting.

Variable	Mean (ms)	Mean HRV per night estimate (SE)	p-value	Mean (ms)	Mean HRV in wake estimate (SE)	p-value	Mean (ms)	Mean HRV in light estimate (SE)	p-value	Mean (ms)	Mean HRV in deep estimate (SE)	p-value
*μ*_0_	40.3	40.32 (2.55)	< 2e-16	54.1	54.13 (1.58)	< 2e-16	44.9	44.93 (1.67)	< 2e-16	35.0	34.98 (1.72)	< 2e-16
*β*_*MG*0 *x*_	47.5	7.16 (2.83)	0.0168	—	—	—	—	—	—	42.2	7.26 (2.43)	0.0054
*β*_*MG*4 *x*_	47.1	6.76 (2.91)	0.0270	—	—	—	—	—	—	—	—	—
*β*_*MG*6 *x*_	54.2	13.87 (2.95)	5.0e-05	66.4	12.22 (3.12)	0.0004	55.1	10.20 (3.30)	0.0040	44.2	9.20 (2.68)	0.0017
	Adjusted R2: 0.4126			Adjusted R2: 0.2972			Adjusted R2: 0.2012			Adjusted R2: 0.2663		
	p-value = 0.0002			p-value = 0.0004			p-value = 0.0040			p-value = 0.0027		

Adjusted mean in milliseconds (ms), mean estimate, standard error (SE) and p-value (Pr > |t-value|). R2: R-squared.

**Table S2.** Univariate series analysis for predicting percent sleep stage by mouth guard setting.

Variable	Mean (%)	Wake estimate ^*bc*^ (SE)	p-value	Mean (%)	Light estimate ^*bc*^ (SE)	p-value
*μ*_0_	15.6	0.52 (0.04)	< 1.9e-14	33.3	0.19 (0.01)	< 2e-16
*β*_*MG*0 *x*_	7.4	-0.12 (0.04)	0.0021	—	—	—
*β*_*MG*4 *x*_	4.6	-0.18 (0.04)	0.0002	39.3	0.05 (0.02)	0.0089
*β*_*MG*6 *x*_	2.7	-0.24 (0.06)	0.0004	39.0	0.05 (0.02)	0.0185

*βNight*	16.0	0.01 (0.00)	0.0146	—	—	—

	bc: exponent = 0.355			bc: exponent = 1.5		
	Adjusted R2: 0.3310			Adjusted R2: 0.1875		
	p-value = 0.0022			p-value = 0.0123		

Adjusted mean in percent, transformed mean estimate ^*bc*^, standard error (SE) and p-value (Pr > |t-value|). R2: R-squared; bc: Box-Cox transformed variable raised to exponent given in final model. Back-transformation to original units was performed (after adjustments relative to intercept) by taking the nth (exponent) root of estimate.

## Supplementary Material

### Supplementary sleep apnea intervention analyses

Clinical evaluations suggested that the subject has a deviated septum, a small jaw with substantial retrognathia (overbite), and evidence of clenching and grinding her teeth during sleep. The subject was diagnosed with mild obstructive sleep apnea in August 2013 and a mandibular splint (mouth guard or MG) was fabricated as an intervention. The subject then wore the device as ordered by her physician. After an initial adjustment period, the subject was monitored while the splint was advanced to achieve relief of apnea symptoms (mainly snoring). The monitoring took place for 40 nights from 11-10-2013 through 12-19-2013. As previously described the subject’s sleep quality was monitored using the Zeo Monitor. In addition, an Equivital belt (Hidalgo, belt type EQ02) was used to collect the subject’s heart rate and R-R interval (beat-to-beat interval) at 15-second intervals (see Methods and Procedures Section below). The unadjusted MG was denoted as MG0x for analysis purposes. Simply inserting a mouth guard creates vertical displacement of the mouth and jaw and also (by design) some horizontal displacement of the jaw. Subsequent adjustments of 4 turns and 6 turns of screws to advance the jaw were denoted as MG4x and MG6x, respectively. Four nights during the time the MG was considered in the analyses had missing data, three nights had no MG wear and 33 nights had the MG worn at settings 0x (13 days), 4x (11 days), and 6x (9 days).

Respiratory sinus arrhythmia is a coupling of the heart rate to follow the breath rate (cardio-pulmonary coupling, CPC). Measures of CPC are usually collected during NREM (light + deep) sleep, which occurs mainly during the first half of the night. In order to accommodate this biological phenomenon and in an attempt to equalize the amount of the data generated by the 15-second Equivital heart rate collection rate, we used an “end-of-night” truncation of 6 am. The collection period used for data analysis was from first sleep bout to last sleep bout before 6 am. This resulted in a minimal change in sleep stage percentages. For example, the 0 mg Cymbalta mean estimates for percent wake (10.5 percent), light (35.4 percent), deep (22.3 percent) and REM (34.2 percent) stages from Table 5 average 15 percent, 33 percent, 23 percent and 30 percent, respectively, when nights are truncated to 6 am. The truncated data shows decreases in percent light and REM sleep which are more prevalent during end-of-night sleep.

Heart rate variability (HRV) is decreased during sleep apnea as the breath is obstructed. An increase in HRV should be seen when obstructive sleep apnea is treated with mandibular advancement. A commonly used measure of HRV is the standard deviation of the R-R interval in milliseconds (ms), also called SDNN. The histograms of R-R interval average (RR-AVG) and the R-R interval standard deviation (RR-STDEV) when grouped by MG adjustments appeared to be roughly normally distributed. One night appeared to be a significant outlier across HRV observations (Thanksgiving night, 11-28-2013, MG setting = 4x) and was removed from all HRV analyses. Excluding this night had a substantial impact on improving model R ^2^ values, but did not change the overall relationship between HRV and MG settings. We analyzed HRV (RR-STDEV) across the entire night and because HRV might be expected to differ among the different sleep stages, we also analyzed the impact of the MG by sleep stage. Univariate regression analyses were performed as previously described (see Methods and Procedures Section below) and assessments showed that all linear regression assumption requirements were satisfied. Table S1 shows a significant increase in total nightly HRV at the MG0x, MG4x and MG6x advanced settings. Regression coefficients are expressed in the original unit of ms for discussion and treatment effects have been adjusted relative to the intercept. The estimate of the y-intercept ( *μ*_0_) for the model with HRV as the dependent variable suggests that the subject’s HRV was approximately 40ms while wearing no mouth guard. The estimated coefficients for the MG settings as independent variables in the model provide the effect on HRV of the mouth guard. The subject’s mean HRV increased to approximately 47ms (7.16 (SE: 2.83), p = 0.0168), 47ms (6.76 (SE: 2.91), p = 0.0270) and 54ms (13.87 (SE: 2.95), p = 5.0×10 ^-5^) while wearing the mouth guard at the MG0x, MG4x and MG6x settings, respectively, for a total nightly increase of 13.87ms in HRV. The breakdown by sleep stage suggested that compared to no mouth guard, MG6x increased the subject’s mean HRV from approximately 54ms to 66ms (12.22 (SE: 3.12), p = 0.0004) in wake; from approximately 45ms to 55ms (10.20 (SE: 3.30), p = 0.0040) in light and from approximately 35ms to 44ms (9.20 (SE: 2.68), p = 0.0017) in deep sleep stages. Interestingly, there was no effect on HRV during REM sleep. This confirms that the effects of MG use we observed are confined to the stages of sleep (NREM) that we felt were expected. None of the models were improved by adding in either night of the study or day of the week.

We also determined the impact that treating sleep apnea had on our subject’s sleep quality. Table S2 shows the effect of MG setting on sleep stages. Because of the short time period tested at each MG setting and the non-normality of the percent sleep data, the Box-Cox procedure and transformation was performed, and models refit as above. As above, for discussion, the mean estimates presented in Table S2 are derived from the regression coefficients which have been adjusted and back-transformed to give the original unit of percent.

The estimate of the y-intercept ( *μ*_0_) for the model with wake as the dependent variable and estimates of the coefficients for MG settings as independent variables suggest that the subject’s mean percent wake steadily decreased from approximately 15.6 percent (0.52 (SE: 0.04), p < 1.9×10 ^-14^) to 7.4 percent (-0.12 (SE: 0.04), p = 0.0021), 4.6 percent (-0.18 (SE: 0.04), p=0.0002) and 2.7 percent (-0.24 (SE: 0.06), p = 0.0004) during the use of MG0x, MG4x and MG6x, respectively.

The estimate of the y-intercept ( *μ*_0_) for the model with light sleep as the dependent variable and estimates of the coefficients for MG settings as independent variables suggest that the subject’s mean percent light sleep increased from approximately 33.3 percent (0.19 (SE: 0.01), p < 2×10 ^-16^) to 39.3 percent (0.05 (SE: 0.02), p = 0.0089) during the use of MG4x and 39 percent (0.05 (SE: 0.02), p = 0.0185) during the use of MG6x. Interestingly, MG use had no significant impact on percent deep or percent REM sleep.

Despite the fact that the 0 mg Cymbalta pharmaceutical intervention portion of the study ended in July 2013, we calculated (above) that the subject was in wake approximately 15 percent of the time and in light sleep approximately 33 percent of the time when nights were truncated at 6 am, which matches the y-intercept estimates ( *μ*_0_) for the no mouth guard state of the sleep apnea study performed from November-December 2013 ( Table S2, 15.6 and 33.3 percent, respectively).

Finally, although we were unable to model percent deep and REM sleep during MG wear, the calculated mean percent deep sleep was 23 percent and the calculated mean percent REM sleep was 29 percent for the 40-night period, which also compares well with the averages calculated from the truncated data (23 percent and 30 percent, respectively). These data indicate a very close agreement in measurements despite an interval of several months and the change in season between the two studies and provides a certain comfort level for the purposes of comparing results from the two interventions.

A clinical sleep study (polysomnography) in January 2014 confirmed the subject to be apnea-free. Ultimately, the final sleep ratios for our subject were “normalized” (wake 2.7 percent; light 39 percent; deep 23 percent; REM 29 percent) by the end of the study (typical values are: wake 5 percent; light 45–55 percent; deep 20–25 percent; REM 25 percent). Note that the final combined average sleep ratios do not quite add up to 100 percent presumably due to errors in estimates for wake and light sleep combined with non-model based values for deep and REM sleep.

### Methods and procedures

***Vital signs.*** The Equivital belt (Hidalgo, belt type EQ02) was used to collect data at 15-second intervals and worn nightly to measure heart rate, breathing rate, and skin temperature. The device has been shown to be reliable for heart rate and R-R interval measures during sleep based on Hidalgo data analysis software quality measures (Equivital software, EQ Manager version 1.1.29.3883). However, clear movement artifacts (R-R interval spikes) could be identified and were removed from the data.

***Heart rate variability analysis.*** Equivital R-R interval data was collected and after movement artifacts were removed R-R interval nightly averages and R-R interval standard deviations were calculated. Movement artifacts were defined as R-R data spikes < 500 ms and > 1100 ms and the artifact data was imputed by filling in the preceding value. Respiratory sinus arrhythmia is a coupling of the heart rate and breath rate (cardio-pulmonary coupling, CPC). Based on coupled autonomic-respiratory oscillations, “stable” sleep shows high frequency coupling (HFC), “unstable” sleep shows low frequency coupling (LFC), while wake and REM sleep show very low frequency coupling (VLFC) ^1^. Therefore, measures of CPC to detect elevated LFC are usually collected during NREM (light + deep) sleep, which occurs mainly during the first half of the night. In order to accommodate this biological phenomenon and in an attempt to equalize the amount of the data generated by the 15-second Equivital heart rate collection rate, we used an “end-of-night” truncation of 6 am. The collection period used for data analysis was from first sleep bout to last sleep bout before 6 am. HRV was defined using the standard deviation of the R-R interval (SDRR), also referred to as the SDNN method (standard deviation of normal-to-normal beats) used by others ^2, 3^. Actiwatch-defined sleep intervals and Zeo-defined first sleep bouts were used to define beginning of sleep and end of night. This was done to normalize behavior after sleep onset. Given that HRV varies with sleep stage, sleep stage transition and time of night, it was important to define an interval that began with sleep onset and ended at the same time every morning.

***Statistics.*** For heart rate variability analysis, the data were collected for 40 consecutive nights beginning from November 10, 2013 to December 19, 2013. Four days of missing data were due to Zeo equipment malfunction or a need to wear alternate head devices. We treated missing data in these analyses as missing as random (MAR). As noted, during this time, the subject wore the mouth guard (MG) for 33 days with settings 0x (13 days), 4x (11 days), and 6x (9 days). The R-R interval data was approximately normal within each MG setting. Variable selection for R-R interval standard deviation (measure of HRV) was performed starting from a full model which included MG setting (0x, 4x, 6x) and manually dropping terms with p-value greater than 0.05. Models were also tested for the impact of study design, days of the week, and model residual diagnosis was assessed as described previously.

**Mouthguard intervention, heart rate variability and sleep response data.**

Date = night of sleep data collection, MGYN = mouthguard wear Yes or No, MGSET = mouthguard setting (0x, 4x, 6x), RRMEANL = Mean of R-R Intervals in Light sleep, RRSTDEVL = Standard Deviation of R-R Intervals in Light sleep, RRMEAND = Mean of R-R Intervals in Deep sleep, RRSTDEVD = Standard Deviation of R-R Intervals in Deep sleep, RRMEANR = Mean of R-R Intervals in REM sleep, RRSTDEVR = Standard Deviation of R-R Intervals in REM sleep, RRMEANW = Mean of R-R Intervals in Wake, RRSTDEVW = Standard Deviation of R-R Intervals in Wake, RRMEANC = Mean of R-R Intervals combined across entire night, RRSTDEVC = Standard Deviation of R-R Intervals combined across entire night, NOMG = No mouthguard wear, MG0x = mouthguard worn at MG0x, MG4x = mouthguard worn at MG4x, MG6x = mouthguard worn at MG6x, HRSLEEP = number of hours of sleep, TPERWAKE = Percent time in Wake when night truncated at 6 am, TPERLIGHT = Percent time in Light sleep when night truncated at 6 am, TPERDEEP = Percent time in Deep sleep when night truncated at 6 am, TPERREM = Percent time in REM sleep when night truncated at 6 am, NIGHT = Night in the study time-course, DAY = Day of the week, DAYCODE = numerical code for day of the week, DAYSUNDAY, DAYMONDAY, etc. = contrast data codes, NA = missing data.
