# Supplementary material for: Normalizing sleep quality disturbed by psychiatric polypharmacy: a single patient open trial (SPOT)
Source: F1000Res. 2016 Sep 5;5:132. Originally published 2016 Feb 3. [Version 2] doi: 10.12688/f1000research.7694.2 (PMC5527988; doi:10.12688/f1000research.7694.2)
Supplement: Peer review history

# Peer review: Referee response for version 2

**DOI:** 10.5256/f1000research.9984.r24049

**Published:** 2017-07-25

**Authors:** Catherine J. Nikles, Thomas A. Harris

**Affiliations:** 1 Center for Clinical Research, The University of Queensland, Brisbane, QLD, Australia

**Background**

 The area of the study is highly relevant to sleep medicine and addresses the compounding factors of polypharmacy in a single case study design. 

 The pathology of SAD is well explained with all of the major aetiologies explored (chronobiological and neurobiology and in-utero environment examined). The individual approach of treating each person’s with SAD is outlined – the background discussion is very interesting and relevant to personalised medicine. All current treatment approaches have been discussed so to have the complexities with varying (on-off”) nature of SAD. 

 Difference between first and second generation anti-depressants are discussed in relevance to the impact on sleep quality and sleep architecture. The authors have highlighted this very important fact that SSRIs and SNRs used to treat SAD typically cause sleep issues. This ultimately leads to polypharmacy to treat SAD and sleep related issues.

**Methodology**

 The methodology to determine sleep and activity monitoring is a well validated tool against the gold standard to study sleep - polysomnography. Descriptive statistics are appropriate in this study and report all appropriate information. A univariate regression model was used to determine all important parameters relevant to sleep (including wake, light, deep and REM). This model is appropriate for the study as it provides clear information on the changes in each of the major sleep patterns in response to the trials run for this single case experimental design.

**Results of the study**

 The result of the study highlights the fundamental importance of personalised medicine and should be published. They have proven in this polypharmacy patient that Cymbalta at therapeutic (60mg) and sub-therapeutic (30mg doses) compromised the participants sleep quality. Subsequent trials identified that no drug trial showed the following:

- Reduced sleep wake cycle
- Reduced light sleep
- Increase in deep sleep
- Increase time in REM sleep.

Because of these trials the study identified an underlying sleep apnea issue with this patient – again supporting the methodology used in this study and the benefits of these trials to improve patient health and outcome.

**Discussion**

 The discussion is well balanced and is contrasted to current findings in the literature. Limitations of the study have been well explored. The authors have explored that washout periods were not used in this design, they limited the number of times any one drug combination was provided – Agree with this approach. To-date there are no other studies that could have been included in this section of the paper to support the findings.

 The only real criticism was the structure - the HRV data and sleep apnea was at the very end of the article. I wonder if this could be early in the paper - this really highlighted the significance of the n-of-1 trial being able to uncover this finding in the patient. It seems that the way this information is presented could be improved.

We have read this submission. We believe that we have an appropriate level of expertise to confirm that it is of an acceptable scientific standard.

# Peer review: Referee response for version 2

**DOI:** 10.5256/f1000research.9984.r16094

**Published:** 2016-12-12

**Authors:** Wilson D. Pace

**Affiliations:** 1 DARTNet Institute, Aurora, CO, USA

This version has been cleaned up considerably but is still long. Removing the sleep apnea sections and cleaning up most of the issues between background, methods, results and discussion helps improve the ability to read the document cleanly. Overall the concept of detailed analysis of the effects of various medications on sleep for an individual patient is a testament to perseverance and a desire for knowledge. The rigorous approach to a clinical evaluation is impressive.

 The results are still hampered by no consideration of carry over effects from one time frame to another. The authors indicate that given the long half-life and perhaps even longer carry-over effect of duloxetine that a pure “washout” would not be practical or possible. This is understood but there are other ways to handle “washout” concerns in N of 1 trials. The most common way is for the data to be removed from analysis during the “cross-over” period. Rarely are pure "placebo" washout periods used. In this report deleting the data from periods of time that a previous drug or level of drug would still be impacting the sleep results is not likely to alter the results from time period to time period. It would likely result in some of the “study” periods being dropped entirely as all or most of the data during one or two periods during the step down of duloxetine would likely be in this window.  The section on not being able to conduct a “treatment washout period” should be revised to indicate that a decision was made to not statistically create a washout period in analyzing the data and the reason this approach was not utilized.

 The reference to “SPOT” N of 1 trials is not available for review. Though a step-down single testing period is considered a N of 1 in this review from a review of the table of contents, the SPOT study approach was not recognized as a N of 1 approach in the AHRQ contracted series of white papers reviewing the area. Be that as it may, the reasonably rigorous approach to studying medication effects on sleep in a complex clinical situation indicates what is possible with a highly motivated patient and diligent care team. Even without the statistical analysis the graphs of the sleep results are compelling that the changes are a result of changes in medications over time and are useful in making clinical decisions, the main point of the activities.

 The term “brain-zap” is not a clinical term and, in fact, does not even appear as a lay term during an internet search. The term should be removed and replaced with a clinical term and referenced. Likewise the term “brain fog” and “jet lagged” are not clinical terms but at least both come up when searched upon.  The concepts should be described in clinical terms and the existence of the phenomena should be referenced. 

 The authors should carefully read through the current version and remove all adjectives and adverbs that do not add scientific value to a sentence. The manuscript has many of these scattered throughout. Some examples are listed below:

 “Treatment  for  SAD  and  its  sequelae  are  also  compounded for peri- and post-menopausal females – a fact which may be under-appreciated in the primary care setting.”

 Either reference this statement as being specific to primary care versus other settings or make the statement more generalized.

 “Prescribing sleep medications to aid in depression-related symptoms in peri- or post-menopausal women that may be susceptible,or have, sleep apnea is therefore highly problematic.”

 The term “highly” either needs to be documented or better yet just removed it adds little.

 Reference the comment that first generation anti-depressants partially exert their effects through restoring sleep. While the drugs have and are used to help with sleep disturbances at low doses it is not clear that the sleep effects have been clearly related to their effects on depression.

   

 "Thus, the resulting polypharmacy used to treat SAD is usually pursued without regard to the timing or dosage of the drugs or concern for drug-drug interactions."

 The word “usually” adds nothing and is not documented/referenced.

 "In order to combat these issues, the management of SAD and related psychiatric disorders should, as noted, be pursued in a more patient-specific or ‘personalized’ manner – something that might not be accomplished at the level of a primary care provider."

 In fact the approachs used in this manuscript are not used widely in any setting that this reviewer is aware of – why single out primary care as the issue?

 These examples were pulled just from the background section. All sections should be reviewed for similar extraneous words that detract from the clinical message.

I have read this submission. I believe that I have an appropriate level of expertise to confirm that it is of an acceptable scientific standard.

# Peer review: Referee response for version 1

**DOI:** 10.5256/f1000research.8286.r12973

**Published:** 2016-05-11

**Authors:** Wilson D. Pace

**Affiliations:** 1 DARTNet Institute, Aurora, CO, USA

This manuscript purports to report on an N-of-1 trial to select therapy for a patient with seasonal affective disorder, sleep apnea and polypharmacy. The outcome of the activities was improved sleep quality. The condition, the treatments to be considered and the outcomes all appear to be excellent concepts to submit to N-of-1 trials. The interventional approaches are described in detail. The medication trial is the closest this process comes to a true N-of-1 trial. This said, the entire manuscript appears to be an early draft that requires extensive rework.

This appears to be a complex case study with some quasi-experimental components of the various intervention approaches. The current version of the manuscript mixes the background with the methods section, the methods section with the results section and the discussion covers interventions outside of the medication trials or the manuscript in general. Again, the medication trial is the only part of the process that approaches an N-of-1 trial and should be the focus of the manuscript.

The current manuscript is very long and difficult to follow. The current draft is just under 10,000 words for the primary paper, excluding the abstract, supplemental material and references. This manuscript would be much easier to follow and comprehend if cut to approximately 3500-4000 words, which is already long for a medical article. This will require extensive editing and decisions about what to include and what to exclude. This reviewer cannot provide full editing guidance but the authors need to consult with others that can help craft future versions.

Areas that need to be addressed:

The extensive description of the patient can be markedly reduced. Further this component of the manuscript should be in the Methods section as it is essentially a description of the study population. The description should focus on the state of affairs just prior to initiating the medication trial. The remaining background is essentially irrelevant to this case study.  The closest part of the therapeutic process that approaches an N-of-1 trial is the medication component. This reviewer recommends focusing on this component of the work if the paper is to be retained as an N-of-1 trail. With this change all the interventions that are discussed prior to or outside of this set of interventions can be dropped and included as the state of the study participant at the start of the trial. The extensive discussion of measurement activities needs to significantly cut and measurement approaches referenced from other literature. The discussion of the measurement approaches is also included in the results section as well as the methods section. Some of the methods section related to the sleep apnea treatment intervention appear to be results in the current draft. This can be solved by dropping the extensive discussion of the sleep apnea diagnosis and intervention entirely as it was not an N-of-1 trial in any sense.

The methods should discuss the N-of-1 approach that was used. The decision to not blind medications should be justified. The cross over pattern selection should discussed. It appears the number of crossovers for each treatment option is limited. This is the primary reason this manuscript appears to be more a case study than a true experimental approach. The number of crossovers should be justified, especially for those medications options that were only studied one time. It appears that the medications were studied primarily in a series of reductions in dosages until the final dosages where there was repeat testing in a back and forth pattern. N-of-1 crossover patterns should be randomized, thus the testing pattern needs to be justified. Given the high variability in sleep quality from night to night the decision to use a limited number of cross-overs seems even more troublesome. The reason for not considering a washout period between treatments needs to be justified.

The discussion of serum markers for major depression disorder is not related to the study methods or results and should be removed. Limitations related to the limited number of crossovers is not discussed. The interference of the mouth guard intervention with the medication trial further complicates the low number of drug crossovers.

This manuscript requires major editing and rewriting prior to being reconsidered.

I have read this submission. I believe that I have an appropriate level of expertise to state that I do not consider it to be of an acceptable scientific standard, for reasons outlined above.

# Response

**Published:** 2016-07-29

**Authors:** Victoria Magnuson

**Affiliations:** J Craig Venter Institute, USA

We would like to thank Dr. Wilson Pace for his insightful review of our manuscript and his many constructive suggestions for improvement. We have amended our manuscript in response and believe it is a much better article as a result.
